# ALPHLARD-NT: Bayesian Method for Human Leukocyte Antigen Genotyping and Mutation Calling through Simultaneous Analysis of Normal and Tumor Whole-Genome Sequence Data

**DOI:** 10.1089/cmb.2018.0224

**Published:** 2019-09-05

**Authors:** Shuto Hayashi, Takuya Moriyama, Rui Yamaguchi, Shinichi Mizuno, Mitsuhiro Komura, Satoru Miyano, Hidewaki Nakagawa, Seiya Imoto

**Affiliations:** ^1^ Human Genome Center, The Institute of Medical Science, The University of Tokyo, Tokyo, Japan.; ^2^ Department of Health Sciences, Faculty of Medical Sciences, Kyushu University, Fukuoka, Japan.; ^3^ RIKEN Center for Integrative Medical Sciences, Tokyo, Japan.; ^4^ Health Intelligence Center, The Institute of Medical Science, The University of Tokyo, Tokyo, Japan.

**Keywords:** Bayesian model, HLA genotyping, HLA mutation calling, whole-exome sequencing, whole-genome sequencing

## Abstract

**Human leukocyte antigen (HLA) genes provide useful information on the relationship between cancer and the immune system. Despite the ease of obtaining these data through next-generation sequencing methods, interpretation of these relationships remains challenging owing to the complexity of HLA genes. To resolve this issue, we developed a Bayesian method, ALPHLARD-NT, to identify HLA germline and somatic mutations as well as HLA genotypes from whole-exome sequencing (WES) and whole-genome sequencing (WGS) data. ALPHLARD-NT showed 99.2% accuracy for WGS-based HLA genotyping and detected five HLA somatic mutations in 25 colon cancer cases. In addition, ALPHLARD-NT identified 88 HLA somatic mutations, including recurrent mutations and a novel HLA-B type, from WES data of 343 colon adenocarcinoma cases. These results demonstrate the potential of ALPHLARD-NT for conducting an accurate analysis of HLA genes even from low-coverage data sets. This method can become an essential tool for comprehensive analyses of HLA genes from WES and WGS data, helping to advance understanding of immune regulation in cancer as well as providing guidance for novel immunotherapy strategies.**

## 1. Introduction

Human leukocyte antigen (HLA) genes are essential components of the immune system, which present peptides to immune cells to facilitate recognition of nonself antigens. HLA genes must be highly polymorphic to effectively carry out this function, with many types or alleles recognized, resulting in high individual variation in immune responses. Therefore, HLA genotyping, in which the specific pair of HLA types is identified for each HLA locus, is essential to understand the immune system. Recently, the interaction between cancer and the immune system has attracted attention (Grivennikov et al., 2010; Schreiber et al., 2011; Kreiter et al., 2015; Rooney et al., 2015; Marty et al., 2017), and somatic mutations in HLA genes have been shown to accumulate in specific cancer types (The Cancer Genome Atlas Research Network, 2014; Testoni et al., 2015; The Cancer Genome Atlas Network, 2015; Giannakis et al., 2016; McGranahan et al., 2017). Therefore, HLA genotyping can further help to understand the link between cancer and immunity, which would benefit personalized medicine.

There are several approaches currently available for HLA genotyping. Conventional approaches use polymerase chain reaction-based methods with sequence-specific oligonucleotides (Saiki et al., 1986), sequence-specific primers (Olerup and Zetterquist, 1992), and sequence-based typing (Santamaria et al., 1992); however, these methods are time consuming and labor intensive, and can only provide information on targeted HLA genes. New methods for HLA genotyping have been developed more recently with advances in molecular techniques, including whole-exome sequencing (WES), whole-genome sequencing (WGS), and RNA sequencing (Boegel et al., 2012; Warren et al., 2012; Kim and Pourmand 2013; Liu et al., 2013; Bai et al., 2014; Szolek et al., 2014; Nariai et al., 2015; Shukla et al., 2015; Dilthey et al., 2016; Xie et al., 2017; Hayashi et al., 2018; Lee and Kingsford, 2018). With these methods, information of both somatic mutations and HLA genotypes can be obtained from the entire sequence, which can facilitate investigations on the relationship between cancer and the immune system. In particular, methods that can specifically call germline or somatic mutations in HLA genes (Shukla et al., 2015; Hayashi et al., 2018; Lee and Kingsford, 2018) are valuable, since these mutations have potential to change immune responses, including tumor immune escape. However, the low coverage of WGS data makes it challenging to detect HLA germline and somatic mutations.

Previously, we developed a Bayesian model, called ALPHLARD (Hayashi et al., 2018), which identifies HLA genotypes and germline mutations from WGS data. ALPHLARD can also call HLA somatic mutations by comparing HLA sequences determined from normal and tumor samples. However, the specificity of the HLA somatic mutation calling is insufficient because ALPHLARD conducts the analyses of normal and tumor samples independently. To resolve this issue, we extended ALPHLARD to construct a new model named ALPHLARD-NT for accurately identifying both HLA germline and somatic mutations as well as HLA genotypes from WGS data. ALPHLARD-NT was validated from WES and WGS data sets from 343 and 25 colon cancer samples, respectively, which demonstrated its good performance in HLA genotyping, along with the ability to call HLA germline and somatic mutations, even from low-coverage data.

## 2. Methods

### 2.1. Human leukocyte antigen reference data

We used the IPD-IMGT/HLA Database (Robinson et al., 2015) as HLA reference sequences in our method. Since the database provides incomplete sequences for most HLA types, we replaced the unknown bases with those of the most similar HLA type. To this end, similarity was determined by measuring the hamming distance in multiple sequence alignments (MSAs) across HLA types obtained from the IPD-IMGT/HLA Database. We used the Allele Frequency Net Database (González-Galarza et al., 2015) for prior information on HLA type frequencies.

### 2.2. Human leukocyte antigen read filtering and realignment

Filtering of HLA reads must be carefully performed for various reasons. First, it is insufficient to use only a human genome reference such as GRCh37 or GRCh38 owing to the high polymorphism of HLA genes. Therefore, a specific HLA database is required, such as the IPD-IMGT/HLA Database. Second, HLA genes and pseudogenes are paralogs and are, therefore, quite similar. Hence, when performing HLA genotyping, it is essential to distinguish reads from an HLA gene of interest from those of other HLA genes and pseudogenes.

In our HLA genotyping pipeline, a BAM file whose reference is the human genome is used as input data. First, sequence reads in the BAM file are filtered by extracting the HLA region, which is defined by chr6:28,477,797–33,448,354 for GRCh37 and chr6:28,510,120–33,480,577 for GRCh38, and covers the HLA-A, -B, -C, -DPA1, -DPB1, -DQA1, -DQB1, and -DRB1 genes. Next, the extracted reads are mapped to all HLA reference sequences using BWA-MEM (version 0.7.17) with the option to obtain information on all identified alignments. Each read is classified based on whether or not the HLA genes produced the read, and if so, which specific gene was involved. This classification is made using alignment scores, which we call HLA read scores (HR scores), and are calculated as follows. Let *x_i_* be the ${i^{{ \rm{th}}}}$ read pair that consists of two single reads ${x_{i , 0}}$ and ${x_{i , 1}}$. In the case of single-end sequence data, *x_i_* consists of one read, ${x_{i , 0}}$. In addition, *t_k_* is defined as the ${k^{{ \rm{th}}}}$ HLA type. If the read ${x_{i , j}}$ is unmapped to the HLA type *t_k_*, then the HR score ${s_{i , j , k}}$ for ${x_{i , j}}$ and *t_k_* is $- \infty$. Otherwise, ${ \tilde x_{i , j , k}}$ and ${ \tilde t_{i , j , k}}$ are the aligned sequences of ${x_{i , j}}$ and *t_k_*, while ${ \tilde x_{i , j , k , n}}$ and ${ \tilde t_{i , j , k , n}}$ are the ${n^{{ \rm{th}}}}$ bases or gaps of ${ \tilde x_{i , j , k}}$ and ${ \tilde t_{i , j , k}}$, respectively. Moreover, the mismatch probability ${ \tilde q_{i , j , k , n}}$ of ${ \tilde x_{i , j , k , n}}$ and ${ \tilde t_{i , j , k , n}}$ can be calculated by

\begin{align*}
 { \tilde q_ { i , j , k , n } } { = 10^ { - { \frac { { { \tilde b } _ { i , j , k , n } } }  { 10 } } } } ,
\end{align*}

where ${ \tilde b_{i , j , k , n}}$ is the Phred base quality of ${ \tilde x_{i , j , k , n}}$. Using the aforementioned definitions, the HR score ${s_{i , j , k}}$ is given by

\begin{align*}
{s_{i , j , k}} = \mathop \sum \limits_n ( s_{i , j , k , n}^{ ( {\tt r} ) } + s_{i , j , k , n}^{ ( {\tt p} ) } ) ,
\end{align*}

where

\begin{align*}
s_{i , j , k , n}^{ ( {\tt r} ) } = \begin{cases} \begin{matrix}{{ \alpha ^{ ( {\tt r} ) }}} & {{ \rm{ ( if}} \;{{ \tilde x}_{i , { \kern 1pt} j , { \kern 1pt} k , { \kern 1pt} n}} \in {B^{{ \rm{ ( }}{\tt N}{ \rm{ ) }}}}{ \rm{ ) }}} \\ 0 \hfill & {{ \rm{ ( if}} \;{{ \tilde x}_{i , { \kern 1pt} j , { \kern 1pt} k , { \kern 1pt} n}}{ \rm{ = }} - { \rm{ ) }}} \hfill \end{matrix}  ,\end{cases}
\end{align*}

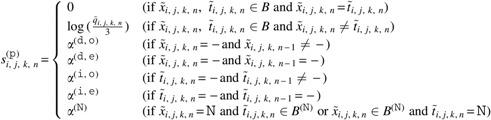


Here, $B = \{  {\tt A} , {\tt C} , {\tt G} , {\tt T} \}$ and ${B^{ ( {\rm N} ) }} = \{  {\tt A} , {\tt C} , {\tt G} , {\tt T} , {\tt N} \}$. $s_{i , j , k , n}^{ ( {\tt r} ) }$ is a reward for the length of the read, and ${ \alpha ^{ ( {\tt r} ) }}$ is a positive hyperparameter for one base. By contrast, $s_{i , j , k , n}^{ ( {\tt p} ) }$ is a penalty for mismatches between the read and the HLA type, and ${ \alpha ^{ ( {\tt d} , {\tt o} ) }}$, ${ \alpha ^{ ( {\tt d} , {\tt e} ) }}$, ${ \alpha ^{ ( {\tt i} , {\tt o} ) }}$, ${ \alpha ^{ ( {\tt i} , {\tt e} ) }}$, and ${ \alpha ^{ ( {\tt N} ) }}$ are negative hyperparameters for deletion opening, deletion extension, insertion opening, insertion extension, and an unknown base N in the read or the HLA type, respectively.

Then, for each read pair *x_i_* and each HLA locus *l*, the score $s_{i , l}^*$ is defined by

\begin{align*}
s_{i , l}^* = \mathop \sum \limits_j { \kern 1pt} \mathop { \max } \limits_{k:{t_k} \in {T_l}} \;{s_{i , j , k}} ,
\end{align*}

where *T_l_* is a set of HLA types of the HLA locus *l*. When *x_i_* is a paired-end read, it is used for genotyping the HLA locus *l* if the following two criteria are satisfied:

\begin{align*}
s_{i , l}^* > { \theta ^{ ( {\tt p} , {\tt s} ) }} ,
\end{align*}

\begin{align*}
s_{i , l}^* - \mathop { \max } \limits_{l \prime \ne l} \;s_{i , l \prime }^* > { \theta ^{ ( {\tt p} , {\tt d} ) }} ,
\end{align*}

Here, ${ \theta ^{ ( {\tt p} , {\tt s} ) }}$ is a hyperparameter of a threshold for the maximum HR score of the locus and ${ \theta ^{ ( {\tt p} , {\tt d} ) }}$ is a hyperparameter of a threshold for the difference between the maximum HR scores of the locus and other loci. However, if *x_i_* is a single-ended read, different thresholds are used; in other words, *x_i_* is used for genotyping the HLA locus *l* if

\begin{align*} 
& s_{i , l}^* > { \theta ^{ ( {\tt s} , {\tt s} ) }} , \\ &s_{i , l}^* - \mathop { \max \;} \limits_{l \prime \ne l} s_{i , l \prime }^* > { \theta ^{ ( {\tt s} , {\tt d} ) }}.
\end{align*}

The former criterion is necessary to collect reads that are likely to be produced by the locus, whereas the latter criterion is needed to exclude reads that might be produced by other loci.

Next, all of the read pairs that satisfy the conditions are realigned to the MSAs of the HLA types of the HLA locus *l*. Realignment of the read ${x_{i , j}}$ is performed using the best HLA type whose index is given by

\begin{align*}
{k^*} = \mathop { \arg \;{\rm max}} \limits_{k:{t_k} \in {T_l}} \;{s_{i , j , k}} ,
\end{align*}

and the realigned read ${ \hat x_{i , j}}$ is obtained by aligning ${x_{i , j}}$ to the MSA ${ \hat t_{{k^*}}}$ of the HLA type ${t_{{k^*}}}$ to match the alignment $( { \tilde x_{i , j , {k^*}}} , \;{ \tilde t_{i , j , {k^*}}} )$. This is done by simply translating the positions of bases and gaps in ${ \tilde t_{i , j , {k^*}}}$ into those in ${ \hat t_{{k^*}}}$.

### 2.3. Bayesian model for human leukocyte antigen analysis

**FIG. 1.**
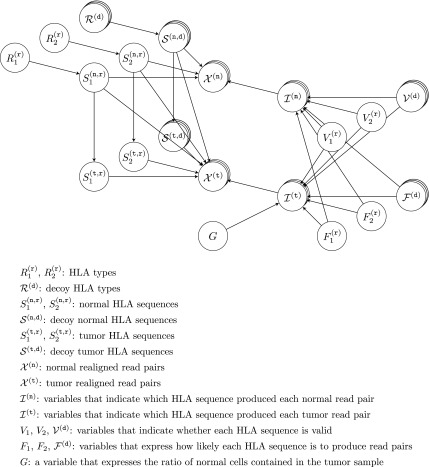
Graphical representation of our method.

We applied a Bayesian model for HLA genotyping and HLA somatic mutation detection, with basically the same structure as our previous method (Hayashi et al., 2018) except for some additional parameters. Figure 1 shows the graphical model. Hereafter, we suppose that the sequence reads are paired-ended for simplicity, and the model for single-ended sequence reads is the same except that the reads are unpaired.

Input data of the model include both the normal and tumor realigned reads. Let $x_i^{ ( {\tt n} ) } = ( x_{i , 0}^{ ( {\tt n} ) } , x_{i , 1}^{ ( {\tt n} ) } )$ be the ${i^{{ \rm{th}}}}$ normal realigned read pair, and $x_i^{ ( {\tt t} ) } = ( x_{i , 0}^{ ( {\tt t} ) } , x_{i , 1}^{ ( {\tt t} ) } )$ be the ${i^{{ \rm{th}}}}$ tumor realigned read pair, where ${\tt n}$ and ${\tt t}$ indicate parameters for the normal and tumor sample, respectively. For each $s \in \{  {\tt n} , {\tt t} \}$, we define $x_{i , j , n}^{ ( s ) }$ as the ${n^{{ \rm{th}}}}$ base of $x_{i , j}^{ ( s ) }$, and $q_{i , j , n}^{ ( s ) }$ as the mismatch probability of $x_{i , j , n}^{ ( s ) }$. Note that the first position of each realigned read is not the beginning of the read but rather that of the MSAs, and $x_{i , j , n}^{ ( s ) }$ and $q_{i , j , n}^{ ( s ) }$ are undefined if the ${n^{{ \rm{th}}}}$ position is not covered by the read. We define $r_{i , j}^{ ( s ) }$ as a set of positions covered by the read $x_{i , j}^{ ( s ) }$ and $r_i^{ ( s ) }$ as $( r_{i , 0}^{ ( s ) } , r_{i , 1}^{ ( s ) } )$.

We denote HLA types of the sample by $R_1^{ ( {\tt r} ) }$ and $R_2^{ ( {\tt r} ) }$, normal HLA sequences by $S_1^{ ( {\tt n} , {\tt r} ) }$ and $S_2^{ ( {\tt n} , {\tt r} ) }$, and tumor HLA sequences by $S_1^{ ( {\tt t} , {\tt r} ) }$ and $S_2^{ ( {\tt t} , {\tt r} ) }$. Here, the sequences of $R_1^{ ( {\tt r} ) }$ and $R_2^{ ( {\tt r} ) }$ are the MSAs of the HLA types. $S_1^{ ( {\tt n} , {\tt r} ) }$ and $S_2^{ ( {\tt n} , {\tt r} ) }$ are used to consider germline variants in $R_1^{ ( {\tt r} ) }$ and $R_2^{ ( {\tt r} ) }$, and $S_1^{ ( {\tt t} , {\tt r} ) }$ and $S_2^{ ( {\tt t} , {\tt r} ) }$ are used to reflect somatic mutations. We also introduce decoy HLA types $R_1^{ ( {\tt d} ) } , \ldots , R_{{ \nu ^{ ( {\tt d} ) }}}^{ ( {\tt d} ) }$, decoy normal HLA sequences $S_1^{ ( n , d ) } , \ldots , S_{{ \nu ^{ ( {\tt d} ) }}}^{ ( \tt {\tt n} , {\tt d} ) }$, and decoy tumor HLA sequences $S_1^{ ( {\tt t} , {\tt d} ) } , \ldots , S_{{ \nu ^{ ( {\tt d} ) }}}^{ ( {\tt t} , {\tt d} ) }$, where ${ \nu ^{ ( {\tt d} ) }}$ is a hyperparameter of the number of the decoy parameters. These parameters are essential to make a robust inference, because their presence can reduce the influence of misclassified reads at the previous filtering step that were actually produced by other HLA genes or pseudogenes. For convenience, we sometimes use $( {R_1} , {R_2} , {R_3} , \ldots , {R_{{ \nu ^{ ( {\tt d} ) }} + 2}} )$, $( S_1^{ ( {\tt n} ) } , S_2^{ ( {\tt n} ) } , S_3^{ ( {\tt n} ) } , \ldots , S_{{ \nu ^{ ( {\tt d} ) }} + 2}^{ ( {\tt n} ) } )$, and $( S_1^{ ( {\tt t} ) } , S_2^{ ( {\tt t} ) } , S_3^{ ( {\tt t} ) } , \ldots , S_{{ \nu ^{ ( {\tt d} ) }} + 2}^{ ( {\tt t} ) } )$ instead of $( R_1^{ ( {\tt r} ) } , R_2^{ ( {\tt r} ) } , R_1^{ ( {\tt d} ) } , \ldots , R_{{ \nu ^{ ( {\tt d} ) }}}^{ ( {\tt d} ) } )$, $( S_1^{ ( {\tt n} , {\tt r} ) } , S_2^{ ( {\tt n} , {\tt r} ) } , S_1^{ ( {\tt n} , {\tt d} ) } , \ldots , S_{{ \nu ^{ ( {\tt d} ) }}}^{ ( {\tt n} , {\tt d} ) } )$, and $( S_1^{ ( {\tt t} , {\tt r} ) } , S_2^{ ( {\tt t} , {\tt r} ) } , S_1^{ ( {\tt t} , {\tt d} ) } , \ldots , S_{{ \nu ^{ ( {\tt d} ) }}}^{ ( {\tt t} , {\tt d} ) } )$, respectively. In addition, in some cases, $( {S_1} , \ldots , {S_{2{ \nu ^{ ( {\tt d} ) }} + 4}} )$ is used instead of $( S_1^{ ( {\tt n} ) } , \ldots , S_{{ \nu ^{ ( {\tt d} ) }} + 2}^{ ( {\tt n} ) } , S_1^{ ( {\tt t} ) } , \ldots , S_{{ \nu ^{ ( {\tt d} ) }} + 2}^{ ( {\tt t} ) } )$. Similar to the notation for read pairs, ${R_{m , n}}$ and ${S_{m , n}}$ are defined as the ${n^{{ \rm{th}}}}$ base of *R_m_* and *S_m_*, respectively.

Next, let $I_i^{ ( {\tt n} ) }$ and $I_i^{ ( {\tt t} ) }$ be parameters that indicate the specific HLA sequence that produced $x_i^{ ( {\tt n} ) }$ and $x_i^{ ( {\tt t} ) }$, respectively. In other words, $I_i^{ ( s ) } = m$ means that $x_i^{ ( s ) }$ was produced by *S_m_*. Note that $I_i^{ ( {\tt n} ) } \in \{  1 , \ldots , { \nu ^{ ( {\tt d} ) }} + 2 \}$ because tumor HLA sequences cannot produce normal sequence reads, and that $I_i^{ ( {\tt t} ) } \in \{  1 , \ldots , 2{ \nu ^{ ( {\tt d} ) }} + 4 \}$ because the tumor sample might also contain normal cells. $I_i^{ ( s ) }$ is independently generated from a distribution governed by $F_1^{ ( {\tt r} ) } , F_2^{ ( {\tt r} ) } , F_1^{ ( {\tt d} ) } , \ldots , F_{{ \nu ^{ ( {\tt d} ) }}}^{ ( {\tt d} ) }$, *G*, and $V_1^{ ( {\tt r} ) } , V_2^{ ( {\tt r} ) } , V_1^{ ( {\tt d} ) } , \ldots , V_{{ \nu ^{ ( {\tt d} ) }}}^{ ( {\tt d} ) }$. Again, we sometimes use convenient notations of $( {F_1} , {F_2} , {F_3} , \ldots , {F_{{ \nu ^{ ( {\tt d} ) }} + 2}} )$ and $( {V_1} , {V_2} , {V_3} , \ldots , {V_{{ \nu ^{ ( {\tt d} ) }} + 2}} )$ instead of $( F_1^{ ( {\tt r} ) } , F_2^{ ( {\tt r} ) } , F_1^{ ( {\tt d} ) } , \ldots , F_{{ \nu ^{ ( {\tt d} ) }}}^{ ( {\tt d} ) } )$, and ($V_1^{ ( {\tt r} ) } , V_2^{ ( {\tt r} ) } , V_1^{ ( {\tt d} ) } , \ldots , V_{{ \nu ^{ ( {\tt d} ) }}}^{ ( {\tt d} ) }$). *F_m_* is a positive real parameter that expresses the likelihood that a read is produced by $S_m^{ ( {\tt n} ) }$ and $S_m^{ ( {\tt t} ) }$. *G* is also a positive real parameter and expresses the ratio of normal cells contained in the tumor sample. *V_m_* is a tuple $( {V_{m , { \kern 1pt} 1}} , \ldots , {V_{m , N}} )$, where *N* is the length of MSAs and ${V_{m , n}}$ is a parameter of 0 or 1, which indicates whether $S_{m , n}^{ ( {\tt n} ) }$ and $S_{m , n}^{ ( {\tt t} ) }$ are valid, as described in more detail hereunder.

The posterior probability of the parameters is given by

\begin{align*}
&p ( \mathcal{R} , {{ \cal S}^{ ( {\tt n} ) }} , {{ \cal S}^{ ( {\tt t} ) }} , \mathcal{F} , { \cal V} , { \mathcal{I}^{ ( {\tt n} ) }} , { \mathcal{I}^{ ( {\tt t} ) }} \vert {{ \cal X}^{ ( {\tt n} ) }} , {{ \cal X}^{ ( {\tt t} ) }} )\\ &\quad \quad \quad \propto p ( {{ \cal X}^{ ( {\tt n} ) }} \vert {{ \cal S}^{ ( {\tt n} ) }} , { \mathcal{I}^{ ( {\tt n} ) }} ) p ( {{ \cal X}^{ ( {\tt t} ) }} \vert {{ \cal S}^{ ( {\tt n} ) }} , {{ \cal S}^{ ( {\tt t} ) }} , { \mathcal{I}^{ ( {\tt t} ) }} )\\ &\quad \quad \quad \ \ \ \quad \quad \times p ( {{ \cal S}^{ ( {\tt t} ) }} \vert {{ \cal S}^{ ( {\tt n} ) }} ) p ( {{ \cal S}^{ ( {\tt n} ) }} \vert \mathcal{R} ) p ( \mathcal{R} ) \\ &\quad \quad \quad \quad \quad \quad \quad \quad \times p ( { \mathcal{I}^{ ( {\tt n} ) }} \vert \mathcal{F} , { \cal V} ) p ( { \mathcal{I}^{ ( {\tt t} ) }} \vert \mathcal{F} , G , { \cal V} ) p ( \mathcal{F} ) p ( G ) p ( { \cal V} ) ,
\end{align*}

where $$\mathcal{R} = ( {R_1} , \ldots , {R_{{ \nu ^{ ( {\tt d} ) }} + 2}} )$$, $${{ \cal S}^{ ( {\tt n} ) }} = ( S_1^{ ( {\tt n} ) } , \ldots , S_{{ \nu ^{ ( {\tt d} ) }} + 2}^{ ( {\tt n} ) } )$$, $${{ \cal S}^{ ( {\tt t} ) }} = ( S_1^{ ( {\tt t} ) } , \ldots , S_{{ \nu ^{ ( {\tt d} ) }} + 2}^{ ( {\tt t} ) } )$$, $$\mathcal{F} = ( {F_1} , \ldots , {F_{{ \nu ^{ ( {\tt d} ) }} + 2}} )$$, $${ \cal V} = ( {V_1} , \ldots , {V_{{ \nu ^{ ( {\tt d} ) }} + 2}} )$$, $${ \mathcal{I}^{ ( {\tt n} ) }} = ( I_1^{ ( {\tt n} ) } , I_2^{ ( {\tt n} ) } , \ldots )$$, $${ \mathcal{I}^{ ( {\tt t} ) }} = ( I_1^{ ( {\tt t} ) } , I_2^{ ( {\tt t} ) } , \ldots )$$, $${{ \cal X}^{ ( {\tt n} ) }} = ( x_1^{ ( {\tt n} ) } , x_2^{ ( {\tt n} ) } , \ldots )$$, and $${{ \cal X}^{ ( {\tt t} ) }} = ( x_1^{ ( {\tt t} ) } , x_2^{ ( {\tt t} ) } , \ldots )$$.

The likelihoods of sequence read pairs are given by

\begin{align*}
p ( {{ \cal X}^{ ( {\tt n} ) }} \vert {{ \cal S}^{ ( {\tt n} ) }} , { \mathcal{I}^{ ( {\tt n} ) }} ) = \prod \limits_i \prod \limits_j \prod \limits_n \;p ( x_{i , j , n}^{ ( {\tt n} ) } \vert {S_{I_i^{ ( {\tt n} ) } , n}} ) ,
\end{align*}

\begin{align*}
p ( {{ \cal X}^{ ( {\tt t} ) }} \vert {{ \cal S}^{ ( {\tt n} ) }} , {{ \cal S}^{ ( {\tt t} ) }} , { \mathcal{I}^{ ( {\tt t} ) }} ) = \prod \limits_i \prod \limits_j \prod \limits_n \;p ( x_{i , j , n}^{ ( {\tt t} ) } \vert {S_{I_i^{ ( {\tt t} ) } , n}} ) ,
\end{align*}

where


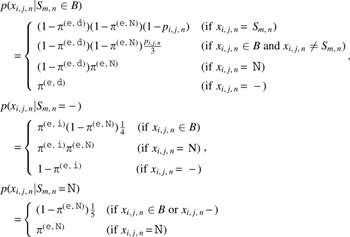


Here, ${ \pi ^{ ( {\tt e} , {\tt d} ) }}$, ${ \pi ^{ ( {\tt e} , {\tt i} ) }}$, and ${ \pi ^{ ( {\tt e} , {\tt N} ) }}$ are hyperparameters of probabilities of a deletion error, insertion error, and ${\tt N}$ in a sequence read, respectively.

The prior probability of tumor HLA sequences is given by

\begin{align*}
p ( {{ \cal S}^{ ( {\tt t} ) }} \vert {{ \cal S}^{ ( {\tt n} ) }} ) = \prod \limits_m \prod \limits_n \;p ( S_{m , n}^{ ( {\tt t} ) } \vert S_{m , n}^{ ( {\tt n} ) } ) ,
\end{align*}

where


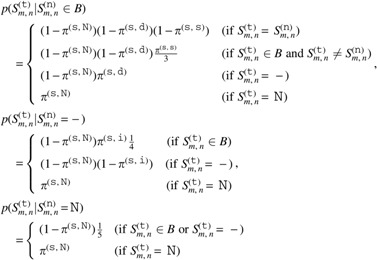


Here, ${ \pi ^{ ( {\tt s} , {\tt s} ) }}$, ${ \pi ^{ ( {\tt s} , {\tt d} ) }}$, ${ \pi ^{ ( {\tt s} , {\tt i} ) }}$, and ${ \pi ^{ ( {\tt s} , {\tt N} ) }}$ are hyperparameters of probabilities of a somatic substitution, somatic deletion, somatic insertion, and ${\tt N}$ in a tumor HLA sequence, respectively.

The prior probability of normal HLA sequences is given by

\begin{align*}
p ( {{ \cal S}^{ ( {\tt n} ) }} \vert \mathcal{R} ) = \bigg( \prod \limits_m \prod \limits_n { \kern 1pt} p \big( S_{m , n}^{ ( {\tt n} , {\tt r} ) } \vert R_{m , n}^{ ( {\tt r} ) } \big) \bigg) \bigg( \prod \limits_m \prod \limits_n { \kern 1pt} p \big( S_{m , n}^{ ( {\rm n} , {\rm d} ) } \vert R_{m , n}^{ ( {\tt d} ) } \big) \bigg) ,
\end{align*}

where


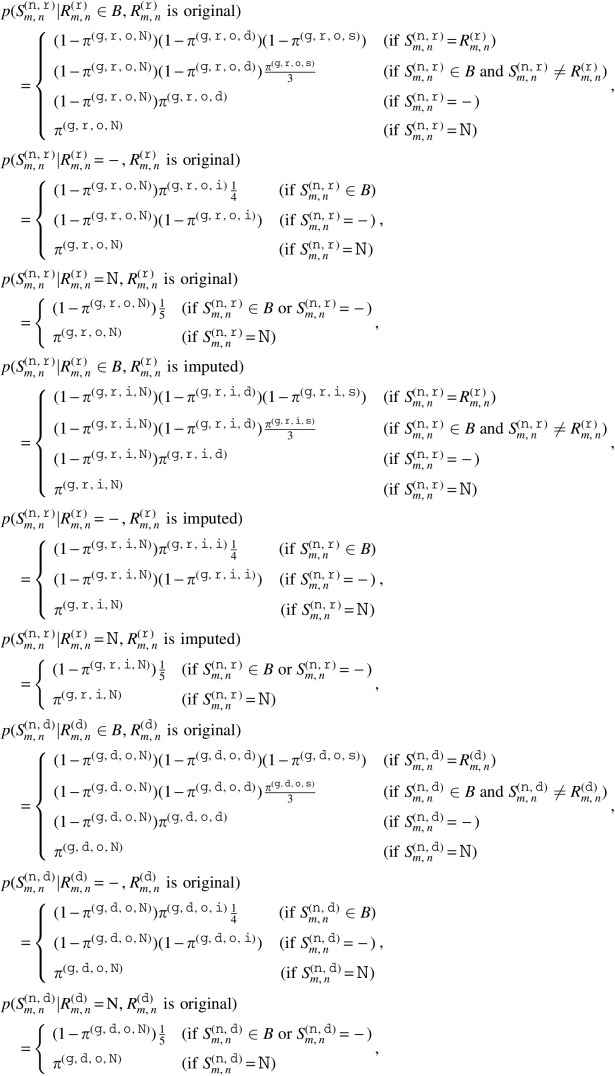



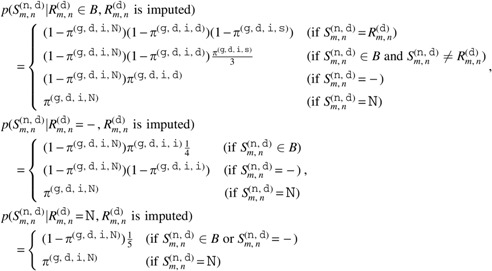


Here, ${ \pi ^{ ( {\tt g} , {\tt r} , {\tt o} , {\tt s} ) }}$, ${ \pi ^{ ( {\tt g} , {\tt r} , {\tt o} , {\tt d} ) }}$, ${ \pi ^{ ( {\tt g} , {\tt r} , {\tt o} , {\tt i} ) }}$, and ${ \pi ^{ ( {\tt g} , {\tt r} , {\tt o} , {\tt N} ) }}$ are hyperparameters of probabilities of a germline substitution, germline deletion, germline insertion, and ${\tt N}$, respectively, in a nondecoy normal HLA sequence at the position where the reference is an original base. The other hyperparameters are also defined in a similar way. The probabilities for an imputed reference base should be larger than those for an original base to reduce the influence of misimputation. In addition, the probabilities for a decoy normal HLA sequence should also be larger than those for a nondecoy normal HLA sequence to achieve robustness against misclassified reads.

The prior probability of HLA types is given by

\begin{align*}
p ( \mathcal{R} ) = \bigg ( \prod \limits_m { \kern 1pt} p \big( R_m^{ ( {\tt r} ) } \big) \bigg ) \bigg( \prod \limits_m { \kern 1pt} p \big( R_m^{ ( {\tt d} ) } \big) \bigg) ,
\end{align*}

where

\begin{align*}
p ( R_m^{ ( {\tt r} ) } = t ) = {p_t} ,
\end{align*}

\begin{align*}
p ( R_m^{ ( {\tt d} ) } ) \propto 1.
\end{align*}

Here, *p_t_* is a prior probability of the HLA type *t*, which was calculated using the Allele Frequency Net Database.

The prior probability of normal indicator variables is given by

\begin{align*}
p ( { \mathcal{I}^{ ( {\tt n} ) }} \vert \mathcal{F} , { \cal V} ) = \prod \limits_i { \kern 1pt} p ( I_i^{ ( {\tt n} ) } \vert \mathcal{F} , { \cal V} ) ,
\end{align*}

where

\begin{align*}
p ( I_i^{ ( {\tt n} ) } = m \vert \mathcal{F} , { \cal V} ) \propto ( \mathop { \max } \limits_{n \in { \cup _j}r_{i , j}^{ ( {\tt n} ) }} {V_m} ) {F_m}.
\end{align*}

This formula means that the read cannot be produced by an HLA sequence without a valid position covered by the read, which is controlled by ${ \cal V}$. Similarly, the prior probability of tumor indicator variables is given by

\begin{align*}
p ( { \mathcal{I}^{ ( {\tt t} ) }} \vert \mathcal{F} , G , { \cal V} ) = \prod \limits_i { \kern 1pt} p ( I_i^{ ( {\tt t} ) } \vert \mathcal{F} , G , { \cal V} ) ,
\end{align*}

where

\begin{align*}
p ( I_i^{ ( {\tt t} ) } = m \in {M^{ ( {\tt n} ) }} \vert \mathcal{F} , G , { \cal V} ) \propto ( \mathop { \max } \limits_{n \in { \cup _j}r_{i , j}^{ ( {\tt t} ) }} {V_m} ) {F_m}G ,
\end{align*}

\begin{align*}
p ( I_i^{ ( {\tt t} ) } = m \in {M^{ ( {\tt t} ) }} \vert \mathcal{F} , G , { \cal V} ) \propto ( \mathop { \max } \limits_{n \in { \cup _j}r_{i , j}^{ ( {\tt t} ) }} { \kern 1pt} {V_{m - ( { \nu ^{ ( {\tt d} ) }} + 2 ) }} ) {F_{m - ( { \nu ^{ ( {\tt d} ) }} + 2 ) }} ,
\end{align*}

\begin{align*}
{M^{ ( {\tt n} ) }} = \{  1 , \ldots , { \nu ^{ ( {\tt d} ) }} + 2 \}  ,
\end{align*}

\begin{align*}
{M^{ ( {\tt t} ) }} = \{  { \nu ^{ ( {\tt d} ) }} + 3 , \ldots , 2{ \nu ^{ ( {\tt d} ) }} + 4 \} \end{align*}

Note that $I_i^{ ( {\tt t} ) } \in {M^{ ( {\tt n} ) }}$ indicates that the read was derived from a normal cell, and $I_i^{ ( {\tt t} ) } \in {M^{ ( {\tt t} ) }}$ indicates that the read was derived from a tumor cell. Furthermore, matched normal-tumor HLA sequences $S_m^{ ( {\tt n} ) }$ and $S_m^{ ( {\tt t} ) }$ share *V_m_* and *F_m_*.

The prior probability of $\mathcal{F}$ is given by

\begin{align*}
p ( \mathcal{F} ) = \bigg( \prod \limits_m { \kern 1pt} p \big( F_m^{ ( {\tt r} ) } \big) \bigg) \bigg( \prod \limits_m { \kern 1pt} p \big( F_m^{ ( {\tt d} ) } \big) \bigg) ,
\end{align*}

where

\begin{align*}
p ( F_m^{ ( {\tt r} ) } ) = \mathcal{L}{ \cal N} ( F_m^{ ( {\tt r} ) } \vert { \mu ^{ ( {\tt f} , {\tt r} ) }} , ( { \sigma ^{ ( {\tt f} , {\tt r} ) }}{ ) ^2} ) ,
\end{align*}

\begin{align*}
p ( F_m^{ ( {\tt d} ) } ) = \mathcal{L}{ \cal N} ( F_m^{ ( {\tt d} ) } \vert { \mu ^{ ( {\tt f} , {\tt d} ) }} , ( { \sigma ^{ ( {\tt f} , {\tt d} ) }}{ ) ^2} )
\end{align*}

Here, $\mathcal{L}{ \cal N}$ is a log-normal distribution, ${ \mu ^{ ( {\tt f} , {\tt r} ) }}$ and ${ ( { \sigma ^{ ( {\tt f} , {\tt r} ) }} ) ^2}$ are hyperparameters of the mean and variance for the nondecoy parameters, and ${ \mu ^{ ( {\tt f} , {\tt d} ) }}$ and ${ ( { \sigma ^{ ( {\tt f} , {\tt d} ) }} ) ^2}$ are hyperparameters of the mean and variance for the decoy parameters. ${ \mu ^{ ( {\tt f} , {\tt d} ) }}$ should be smaller than ${ \mu ^{ ( {\tt f} , {\tt r} ) }}$ because sequence reads mapped to decoy HLA sequences should be removed at the filtering step.

The prior probability of *G* is given by

\begin{align*}
p ( G ) = \mathcal{L}{ \cal N} ( G \vert { \mu ^{ ( {\tt g} ) }} , ( { \sigma ^{ ( {\tt g} ) }}{ ) ^2} ) ,
\end{align*}

where ${ \mu ^{ ( {\tt g} ) }}$ and ${ ( { \sigma ^{ ( {\tt g} ) }} ) ^2}$ are hyperparameters of the mean and variance for normal contamination.

The prior probability of ${ \cal V}$ is given by

\begin{align*}
p ( { \cal V} ) = \bigg( \prod \limits_m \prod \limits_n { \kern 1pt} p \big( V_{m , n}^{ ( {\tt r} ) } \big) \bigg) \; \bigg( \prod \limits_m \prod \limits_n { \kern 1pt} p \big( V_{m , n}^{ ( {\tt d} ) } \vert V_{m , n - 1}^{ ( {\tt d} ) } \big) \bigg) ,
\end{align*}

where


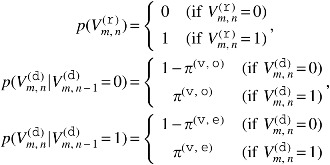


Here, ${ \pi ^{ ( {\tt v} , {\tt o} ) }}$ and ${ \pi ^{ ( {\tt v} , {\tt e} ) }}$ are hyperparameters of probabilities of a validity flag opening and a validity flag extension, respectively. Note that $V_{m , n}^{ ( {\tt r} ) }$ must always be 1.

### 2.4. Markov chain Monte Carlo-based parameter sampling

The parameters are sampled from the Bayesian model using Markov chain Monte Carlo. Gibbs sampling is primarily used to sample all parameters except for *F_m_* and *V_m_*.

A candidate parameter, $F_m^*$, is first sampled using the Metropolis–Hastings algorithm whose proposal distribution is given by

\begin{align*}
F_m^* \sim \mathcal{L}{ \cal N} ( \log {F_m} , ( \sigma _m^{ ( {\tt f} , {\tt p} ) }{ ) ^2} ) ,
\end{align*}

where ${ ( \sigma _m^{ ( {\tt f} , {\tt p} ) } ) ^2}$ is a hyperparameter of the variance of the proposal distribution. The acceptance ratio ${r^*}$ is calculated by

\begin{align*}
 { r^* } = { \frac { p ( { \mathcal { I } ^ { ( { \tt n } ) } } \vert { \mathcal { F } ^* } , { \cal V } ) p ( { \mathcal { I } ^ { ( { \tt t } ) } } \vert { \mathcal { F } ^* } , { \cal V } ) p ( F_m^* ) }  { p ( { \mathcal { I } ^ { ( { \tt n } ) } } \vert \mathcal { F } , { \cal V } ) p ( { \mathcal { I } ^ { ( { \tt t } ) } } \vert \mathcal { F } , { \cal V } ) p ( { F_m } ) } } ,
\end{align*}

where ${ \mathcal{F}^*} = ( {F_1} , \ldots , {F_{m - 1}} , F_m^* , {F_{m + 1}} , \ldots , {F_{{ \nu ^{ ( {\tt d} ) }} + 2}} )$. A candidate parameter, $V_m^*$, is sampled using the Metropolis–Hastings algorithm whose proposal distribution is analogous to the Wolff algorithm (Wolff, 1989), which is used for sampling of the Ising model. $V_m^*$ is generated by Algorithm 1. Then, ${ \mathcal{I}^{ ( {\tt n} ) *}}$ and ${ \mathcal{I}^{ ( {\tt t} ) *}}$ are also sampled using Gibbs sampling given $V_m^*$. The acceptance ratio ${r^*}$ is calculated by

\begin{align*} 
{ r^* } &= { \frac { \prod \nolimits_ { n = 1 } ^ { r + 1 } { p ( V_ { m , n } ^* \vert V_ { m , n - 1 } ^* ) } }  { \prod \nolimits_ { n = 1 } ^ { r + 1 } { p ( { V_ { m , n } } \vert { V_ { m , n - 1 } } ) } } } \\ &\quad \quad \times { \frac { { { ( \pi _v^ { ( { \tt v } , { \tt p } ) } ) } ^ { r - l } } { { ( 1 - \pi _v^ { ( { \tt v } , { \tt p } ) } ) } ^ { [ l \ne 1 \wedge { V_ { l - 1 } } \ne v ] + [ r \ne N \wedge { V_ { r + 1 } } \ne v ] } } }  { { { ( \pi _ { 1 - v } ^ { ( { \tt v } , { \tt p } ) } ) } ^ { r - l } } { { ( 1 - \pi _ { 1 - v } ^ { ( { \tt v } , { \tt p } ) } ) } ^ { [ l \ne 1 \wedge { V_ { l - 1 } } \ne v ] + [ r \ne N \wedge { V_ { r + 1 } } \ne v ] } } } } 
\end{align*}

\begin{align*}
\quad \quad \quad \quad \times { \frac { p ( { { \cal X } ^ { ( { \tt n } ) } } \vert { { \cal S } ^ { ( { \tt n } ) } } , { { \cal V } ^* } ) p ( { { \cal X } ^ { ( { \tt t } ) } } \vert { { \cal S } ^ { ( { \tt n } ) } } , { { \cal S } ^ { ( { \tt t } ) } } , { { \cal V } ^* } ) }  { p ( { { \cal X } ^ { ( { \tt n } ) } } \vert { { \cal S } ^ { ( { \tt n } ) } } , { \cal V } ) p ( { { \cal X } ^ { ( { \tt t } ) } } \vert { { \cal S } ^ { ( { \tt n } ) } } , { { \cal S } ^ { ( { \tt t } ) } } , { \cal V } ) } } .
\end{align*}

We set $1 - { \pi ^{ ( {\tt v} , {\tt o} ) }}$ and ${ \pi ^{ ( {\tt v} , {\tt e} ) }}$ to $\pi _0^{ ( {\tt v} , {\tt p} ) }$ and $\pi _1^{ ( {\tt v} , {\tt p} ) }$, respectively, so that the acceptance ratio can be calculated by


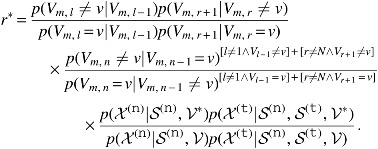


\begin{align*}
\quad \quad \quad \quad \times { \frac { p ( { { \cal X } ^ { ( { \tt n } ) } } \vert { { \cal S } ^ { ( { \tt n } ) } } , { { \cal V } ^* } ) p ( { { \cal X } ^ { ( { \tt t } ) } } \vert { { \cal S } ^ { ( { \tt n } ) } } , { { \cal S } ^ { ( { \tt t } ) } } , { { \cal V } ^* } ) }  { p ( { { \cal X } ^ { ( { \tt n } ) } } \vert { { \cal S } ^ { ( { \tt n } ) } } , { \cal V } ) p ( { { \cal X } ^ { ( { \tt t } ) } } \vert { { \cal S } ^ { ( { \tt n } ) } } , { { \cal S } ^ { ( { \tt t } ) } } , { \cal V } ) } } .
\end{align*}

### 2.5. Efficient sampling from multimodal posteriors

In addition to the standard sampling approaches mentioned earlier, we applied some additional elaborate sampling schemes to prevent the parameters from becoming stuck in a local optimum. One such scheme swaps parts of the nondecoy and decoy HLA sequences. First, a nondecoy index $m \in \{  1 , 2 \}$, decoy index $m \prime \in \{  3 , \cdots , { \nu ^{ ( {\tt d} ) }} + 2 \}$, and interval *i* such that $\forall n \in i; {V_{m \prime , n}} = 1$ are sampled uniformly. Next, $S_{m , n}^{ ( {\tt n} ) }$ and $S_{m \prime , n}^{ ( {\tt n} ) }$, and $S_{m , n}^{ ( {\tt t} ) }$ and $S_{m \prime , n}^{ ( {\tt t} ) }$ are swapped for all $n \in i$. Finally, $R_m^*$, $R_{m \prime }^*$, ${ \mathcal{I}^{ ( {\tt n} ) *}}$, and ${ \mathcal{I}^{ ( {\tt t} ) *}}$ are sampled using Gibbs sampling given ${{ \cal S}^{ ( {\tt n} ) *}}$ and ${{ \cal S}^{ ( {\tt t} ) *}}$, which are the normal and tumor HLA sequences after swapping. Consequently, the acceptance ratio ${r^*}$ is given by

\begin{align*}
 { r^* } = { \frac { p ( { { \cal X } ^ { ( { \tt n } ) } } \vert { { \cal S } ^ { ( { \tt n } ) * } } , { \cal V } ) p ( { { \cal X } ^ { ( { \tt t } ) } } \vert { { \cal S } ^ { ( { \tt n } ) * } } , { { \cal S } ^ { ( { \tt t } ) * } } , { \cal V } ) p ( { { \cal S } ^ { ( { \tt n } ) * } } ) }  { p ( { { \cal X } ^ { ( { \tt n } ) } } \vert { { \cal S } ^ { ( { \tt n } ) } } , { \cal V } ) p ( { { \cal X } ^ { ( { \tt t } ) } } \vert { { \cal S } ^ { ( { \tt n } ) } } , { { \cal S } ^ { ( { \tt t } ) } } , { \cal V } ) p ( { { \cal S } ^ { ( { \tt n } ) } } ) } } .
\end{align*}

This sampling method helps to determine which HLA sequences should be decoys.

Another scheme involves sampling an HLA type and matched normal-tumor HLA sequences simultaneously. For all $m \in \{  1 , \ldots , { \nu ^{ ( {\tt d} ) }} + 2 \}$, $S_m^{ ( {\tt n} , {\tt N} ) }$ and $S_m^{ ( {\tt t} , {\tt N} ) }$ are defined by

\begin{align*} 
S_{m , n}^{ ( {\tt n} , {\tt N} ) } &= \begin{cases} \begin{matrix} {S_{m , n}^{ ( {\tt n} ) }} & {{ \rm{ ( if }}{D_{m , n}}{ \rm{ > 0 ) }}} \\ {\tt N} & {{ \rm{ ( if }}{D_{m , n}}{ \rm{ = 0 ) }}} \\ \end{matrix}, \end{cases} \\ S_{m , n}^{ ( {\tt t} , {\tt N} ) } &= \begin{cases} \begin{matrix}{S_{m , n}^{ ( {\tt t} ) }} & {{ \rm{ ( if }}{D_{m , n}}{ \rm{ > 0 ) }}} \\ {\tt N} & {{ \rm{ ( if }}{D_{m , n}}{ \rm{ = 0 ) }}} \\ \end{matrix},  \end{cases} \\ {D_{m , n}} &= D_{m , n}^{ ( {\tt n} ) } + D_{m , n}^{ ( {\tt t} ) } + D_{m + { \nu ^{ ( {\tt d} ) }} + 2 , n}^{ ( {\tt t} ) } , \\ D_{m , n}^{ ( {\tt n} ) } &= \vert \{  ( i , j ) \vert I_i^{ ( {\tt n} ) } = m , n \in {r_{i , j}} \}  \vert , \\ D_{m , n}^{ ( {\tt t} ) } &= \vert \{  ( i , j ) \vert I_i^{ ( {\tt t} ) } = m , n \in {r_{i , j}} \}  \vert
\end{align*}

In other words, $S_m^{ ( {\tt n} , {\tt N} ) }$ and $S_m^{ ( {\tt t} , {\tt N} ) }$ are basically the same as $S_m^{ ( {\tt n} ) }$ and $S_m^{ ( {\tt t} ) }$, and bases not covered by any read are replaced with Ns. Next, $R_m^*$ is sampled given $S_m^{ ( {\tt n} , {\tt N} ) }$, $S_m^{ ( {\tt n} ) *}$ is sampled given $R_m^*$ and $S_m^{ ( {\tt t} , {\tt N} ) }$, and $S_m^{ ( {\tt t} ) *}$ is sampled given $S_m^{ ( {\tt n} ) *}$ in order. Then, the acceptance ratio ${r^*}$ is given by


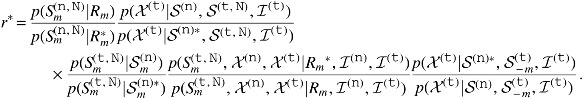


This sampling functions in a similar way to blocked Gibbs sampling of *R_m_*, $S_m^{ ( {\tt n} ) }$, and $S_m^{ ( {\tt t} ) }$. This blocked Gibbs sampling requires substantial computation time because $S_m^{ ( {\tt n} ) }$ and $S_m^{ ( {\tt t} ) }$ must be integrated out for each HLA type. By contrast, our scheme requires much less time because $S_m^{ ( {\tt n} ) }$ and $S_m^{ ( {\tt t} ) }$ are integrated out only for *R_m_* and $R_m^*$.

Other strategies were further used to obtain better parameters. First, reference sequences are periodically copied to HLA sequences. Second, sequence reads are assigned to decoy sequences if there are mismatches between the sequence reads and the reference sequences. These approaches help to reduce the incidence of false-positive mutations and retain only the mutations that seem true. The multistart method is also used to obtain better initial parameters. Moreover, parallel tempering is used to move parameters from mode to mode.

### 2.6. Human leukocyte antigen analysis from sampled parameters

HLA analysis is conducted based on the sampled parameters. HLA genotyping is performed by counting the number of sampled HLA types, and germline or somatic mutations are identified by finding different bases between HLA types and normal HLA sequences, or between normal and tumor HLA sequences, respectively.

## 3. Results

### 3.1. Human leukocyte antigen genotyping from whole-genome sequencing data

We first evaluated the accuracy of this method for HLA genotyping from a WGS data set. For comparison, we applied ALPHLARD-NT, ALPHLARD (Hayashi et al., 2018), and POLYSOLVER (Shukla et al., 2015) to WGS data of 25 colon cancer samples, which were used by Hayashi et al. (2018). The performance comparison is summarized in Table 1. Overall, ALPHLARD-NT outperformed POLYSOLVER at all resolutions for all HLA loci. ALPHLARD-NT also achieved slightly higher accuracy than ALPHLARD because ALPHLARD-NT can use information from both normal and tumor samples, whereas ALPHLARD can only use information from normal samples.

**Table 1.** Comparison of the Accuracy of Whole-Genome Sequencing-Based Human Leukocyte Antigen Genotyping with ALPHLARD-NT, ALPHLARD, and POLYSOLVER

	*ALPHLARD-NT*	*ALPHLARD*	*POLYSOLVER*
HLA-A			
First	**100% (50/50)**	**100% (50/50)**	**100% (50/50)**
Second	**100% (50/50)**	98.0% (49/50)	98.0% (49/50)
Third	**98.0% (49/50)**	**98.0% (49/50)**	90.0% (45/50)
HLA-B			
First	**100% (48/48)**	**100% (48/48)**	91.7% (44/48)
Second	**100% (48/48)**	**100% (48/48)**	85.4% (41/48)
Third	**97.9% (47/48)**	95.8% (46/48)	81.3% (39/48)
HLA-C			
First	**100% (50/50)**	**100% (50/50)**	**100% (50/50)**
Second	**100% (50/50)**	98.0% (49/50)	90.0% (45/50)
Third	**100% (50/50)**	98.0% (49/50)	86.0% (43/50)
HLA-DPA1			
First	**100% (24/24)**	**100% (24/24)**	N/A
Second	**100% (24/24)**	**100% (24/24)**	N/A
Third	**100% (24/24)**	**100% (24/24)**	N/A
HLA-DPB1			
First	**100% (22/22)**	**100% (22/22)**	N/A
Second	**100% (22/22)**	**100% (22/22)**	N/A
Third	**100% (22/22)**	**100% (22/22)**	N/A
HLA-DQA1			
First	**100% (24/24)**	**100% (24/24)**	N/A
Second	**95.8% (23/24)**	**95.8% (23/24)**	N/A
Third	**95.8% (23/24)**	**95.8% (23/24)**	N/A
HLA-DQB1			
First	**100% (18/18)**	**100% (18/18)**	N/A
Second	**94.4% (17/18)**	**94.4% (17/18)**	N/A
Third	**94.4% (17/18)**	**94.4% (17/18)**	N/A
HLA-DRB1			
First	**100% (24/24)**	**100% (24/24)**	N/A
Second	**100% (24/24)**	**100% (24/24)**	N/A
Third	**100% (24/24)**	**100% (24/24)**	N/A
Total			
First	**100% (260/260)**	**100% (260/260)**	97.3% (144/148)
Second	**99.2% (258/260)**	98.5% (256/260)	91.2% (135/148)
Third	**98.5% (256/260)**	97.7% (254/260)	85.8% (127/148)

N/A indicates that the method does not support the HLA locus.

HLA, human leukocyte antigen.

Bold values indicate that the method achieved the highest accuracy for the HLA locus at the resolution.

### 3.2. Detection of human leukocyte antigen mutations from whole-genome sequencing data

We also searched for HLA class I somatic mutations among the WGS data from the 25 colon cancer samples using ALPHLARD-NT, POLYSOLVER, and EBCall (Shiraishi et al., 2013), which is a standard mutation caller. ALPHLARD-NT called one substitution, two insertions, and two deletions, all of which were verified by the TruSight HLA Sequencing Panels (Weimer et al., 2016). All four indels called are known to lead to the loss of function of the HLA alleles, and might contribute to immune escape. However, POLYSOLVER and EBCall detected no and one mutation, respectively, which was likely due to the low coverage of the data set.

### 3.3. Detection of human leukocyte antigen mutations from whole-exome sequencing data

**FIG. 2.**
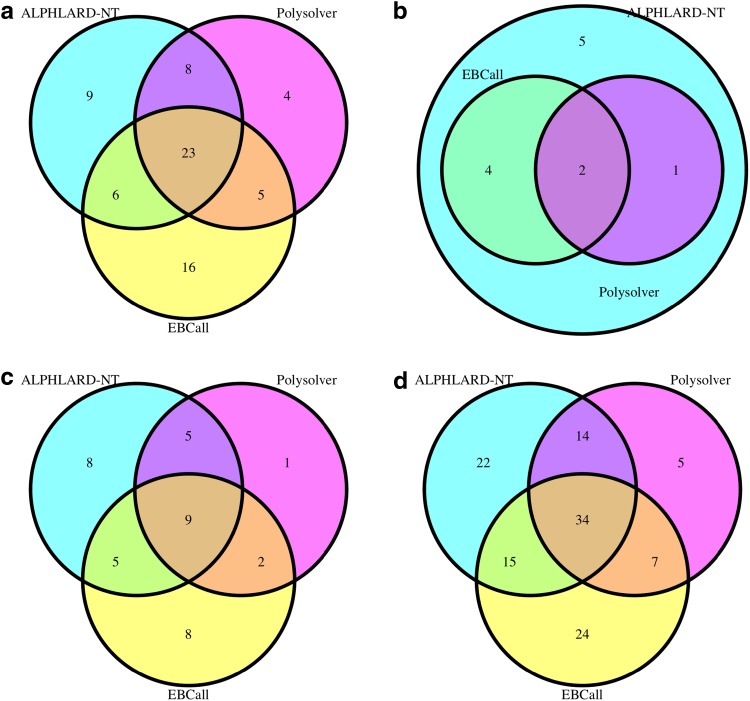
Venn diagrams of the number of HLA somatic mutations identified by ALPHLARD-NT, POLYSOLVER, and EBCall for **(a)** substitutions, **(b)** insertions, **(c)** deletions, and **(d)** all mutations. HLA, human leukocyte antigen.

Next, we applied ALPHLARD-NT, POLYSOLVER, and EBCall to a WES data set of 343 colon adenocarcinoma cases from The Cancer Genome Atlas (TCGA). Figure 2 shows the Venn diagrams of the identified HLA class I somatic mutations with each method. This figure demonstrates the high sensitivity of ALPHLARD-NT (88 mutations) compared with POLYSOLVER (60 mutations) and EBCall (80 mutations), which is especially remarkable for insertions. ALPHLARD-NT detected seven insertions at the beginning of exon 4 of HLA class I genes, which is a known hotspot of indels (Mizuno et al., 2018), whereas POLYSOLVER and EBCall identified no and three insertions at this hotspot, respectively. ALPHLARD-NT also identified 12 deletions at the same position. These recurrent frameshift indels seemed to be positively selected for immune escape caused by loss of function of the HLA alleles.

In addition, ALPHLARD-NT detected a novel HLA-B allele whose exon sequence is the same as HLA-B*35:08:01 except that the 25th base is C rather than G, which changes the 9th amino acid from V to L. The protein produced by the new allele is also novel and not registered in the IPD-IMGT/HLA Database, indicating that the allele defines a new HLA type name at the second field.

**Table:** 

**Algorithm 1** Generate a candidate parameter ${V^<sup>*</sup>}$ using the Wolff algorithm
**Input:**
*V*: the current parameter
*N*: the length of *V*
$\pi _0^{ ( {\tt v} , {\tt p} ) }$: probability for 0-cluster extension
$\pi _1^{ ( {\tt v} , {\tt p} ) }$: probability for 1-cluster extension
**Output:**
$${V^<sup>*</sup>}$$: candidate parameter
1: **function**Wolff$$( V , \; \pi _0^{ ( {\tt v} , {\tt p} ) } , \; \pi _1^{ ( {\tt v} , {\tt p} ) } )$$
2: Sample a position *p* uniformly
3: $$v \leftarrow {V_p}$$
4: $$b \leftarrow p$$
5: **while**$b > 1$**and**${V_{b - 1}} = v$**do**
6: **break** with probability $1 - \pi _v^{ ( {\tt v} , {\tt p} ) }$
7: $$b \leftarrow b - 1$$
8: **end while**
9: $$e \leftarrow p$$
10: **while**$e < N$**and**${V_{e + 1}} = v$**do**
11: **break** with probability $1 - \pi _v^{ ( {\tt v} , {\tt p} ) }$
12: $$e \leftarrow e + 1$$
13: **end while**
14: $${V^<sup>*</sup>} \leftarrow V$$
15: **for**$n \leftarrow b$ to *e***do**
16: $$V_n^<sup>*</sup> \leftarrow 1 - v$$
17: **end for**
18: **return**$${V^<sup>*</sup>}$$
19: **end function**

## 4. Conclusion

In this article, we have presented a new Bayesian method, ALPHLARD-NT, which identifies HLA germline and somatic mutations as well as HLA genotypes. Comparison of the performance of ALPHLARD-NT clearly demonstrated its higher accuracy than existing methods for WGS-based HLA genotyping. ALPHLARD-NT also detected HLA somatic mutations from both WES and WGS data. In general, HLA mutation calling is difficult mainly due to the similarity of HLA genes and pseudogenes. We dealt with this problem by applying sophisticated filtering criteria and using decoy-related parameters that reduced the influence of misclassified reads at the filtering step. Although these approaches work well for HLA class I mutation calling, identification of HLA class II mutations remains a challenge, since databases tend to be relatively incomplete for identifying class II genes and pseudogenes compared with class I genes.

With the continuous accumulation of large amounts of WES and WGS data, HLA mutation calling from these data sets is a fundamental step in cancer immunogenomics. Thus, we expect that our method will be an essential tool for comprehensive analyses of HLA genes from WES and WGS data.
